# Computational Model Explains High Activity and Rapid Cycling of Rho GTPases within Protein Complexes

**DOI:** 10.1371/journal.pcbi.0020172

**Published:** 2006-12-01

**Authors:** Andrew B Goryachev, Alexandra V Pokhilko

**Affiliations:** Systems Biology Group, Bioinformatics Institute, Singapore, Singapore; Weill Medical College of Cornell University, United States of America

## Abstract

Formation of multiprotein complexes on cellular membranes is critically dependent on the cyclic activation of small GTPases. FRAP-based analyses demonstrate that within protein complexes, some small GTPases cycle nearly three orders of magnitude faster than they would spontaneously cycle in vitro. At the same time, experiments report concomitant excess of the activated, GTP-bound form of GTPases over their inactive form. Intuitively, high activity and rapid turnover are contradictory requirements. How the cells manage to maximize both remains poorly understood. Here, using GTPases of the Rab and Rho families as a prototype, we introduce a computational model of the GTPase cycle. We quantitatively investigate several plausible layouts of the cycling control module that consist of GEFs, GAPs, and GTPase effectors. We explain the existing experimental data and predict how the cycling of GTPases is controlled by the regulatory proteins in vivo. Our model explains distinct and separable roles that the activating GEFs and deactivating GAPs play in the GTPase cycling control. While the activity of GTPase is mainly defined by GEF, the turnover rate is a sole function of GAP. Maximization of the GTPase activity and turnover rate places conflicting requirements on the concentration of GAP. Therefore, to achieve a high activity and turnover rate at once, cells must carefully maintain concentrations of GEFs and GAPs within the optimal range. The values of these optimal concentrations indicate that efficient cycling can be achieved only within dense protein complexes typically assembled on the membrane surfaces. We show that the concentration requirement for GEF can be dramatically reduced by a GEF-activating GTPase effector that can also significantly boost the cycling efficiency. Interestingly, we find that the cycling regimes are only weakly dependent on the concentration of GTPase itself.

## Synopsis

A large variety of cellular processes, such as the formation of filopodia or transport vesicles, require that large protein complexes are precisely positioned on intracellular membranes to execute a specific task and then are promptly disassembled to perform their function elsewhere. Small GTPases play a major role in the spatiotemporal control of these complexes. Their function is based on the unique property of cycling between the active GTP-bound state, in which they enable complex formation, and the inactive GDP-bound state, which promotes complex dissolution. Recent experiments based on fluorescence recovery after photobleaching have found that some small GTPases rapidly cycle within protein complexes, causing continuous release and recruitment of the complex components. The seemingly futile cycling is accompanied by a large excess of the active form. This puzzling behavior challenges one's intuition and calls for the application of quantitative methods. Here, Goryachev and Pokhilko use computational modeling to identify regulatory mechanisms that could enable GTPases to cycle with the experimentally observed frequency and efficiency. They show that to achieve high activity and turnover simultaneously, the concentrations of the regulatory molecules that control GTPase cycling should be tightly maintained within the optimal range.

## Introduction

The Ras superfamily of small GTPases [1] has recently emerged as the central element of a variety of molecular modules that provide spatial and temporal control for protein complex formation in the cell [2–4]. The operation of these ubiquitous control modules is based on the intrinsic property of GTPases to cycle between an inactive GDP-bound state (RD) and an active GTP-bound state (RT). The biological function of GTPases is performed only by the active form that binds and activates a broad range of effector proteins [5]. Many of the effectors, such as WASP [6] and CNK1 [7], are scaffold-like proteins that change their conformation and gain the ability to recruit several binding partners while they are associated with the RT. Such effectors serve as platforms for the assembly of functional multiprotein complexes. Once brought together by the GTPase-controlled scaffolds, these complexes perform structural and signaling functions crucial for cell existence, for example, actin polymerization [8,9] and activation of the p38 MAP kinase pathways [7,10].

Since the cellular localization and timing of the existence of these complexes are determined by the local availability of the RT, a simultaneous sharp increase in the local GTPase concentration and the relative abundance of its active form are expected in the hotspots of complex formation. Owing to recent advances in imaging techniques [11], such a highly localized cellular distribution of the RT was indeed observed in several in vivo systems, e.g., for cdc42 in the extending filopodia of fibroblasts [12] and in emerging yeast buds [13]. In the intracellular environment, GTPases are tightly controlled by multiple regulatory proteins. GTPase activating proteins (GAPs) inhibit the GTPase activity by accelerating hydrolysis of GTP into GDP. The activation of GTPases is catalyzed by guanine nucleotide exchange factors (GEFs) that facilitate the replacement of GDP by GTP. One would expect that to control complex formation, it is sufficient to simply segregate the activity of GEFs and GAPs spatially and/or temporally. Some small GTPases indeed appear to follow this strategy. For example, activation of the Arf family of GTPases by their cognate GEFs reversibly changes their conformation to simultaneously enable binding to the effectors and to lipid membranes [14]. Activated GTPases then anchor to a membrane and recruit specific effectors, such as coat proteins COP I and COP II [15]. Once the nascent fragment of coat is solidified by the protein–protein interactions between coatomers, GAPs deactivate Arf GTPases and return their conformation to the binding-incompetent form, thus preparing for the vesicle uncoating and release of coatomers and GTPases back to the cytoplasm [16]. Within this framework, Arf molecules were thought to undergo a single activation cycle per coat formation. Such a mechanism would seem to provide the necessary complex-control function at a low energetic cost (each cycle consumes one GTP molecule).

Therefore, it came as a surprise [17] that some GTPases perform rapid, seemingly futile, cycles of GTP hydrolysis within respective GTPase-controlled complexes. In their seminal work published a decade ago, Zerial and colleagues using biochemical methods discovered that Rab5 vigorously converts GTP into GDP in the absence of vesicle fusion. Recent FRAP-based studies [13,18,19] confirmed this early observation and furthermore demonstrated that, driven by cycling of GTPases, the GTPase-controlled protein complexes rapidly dissolve into the cytoplasm and reassemble on the membrane with a half-life of ∼1–5 s. Only such a dynamic exchange between the free cytoplasmic proteins and the complexes on the membrane can explain the rapid fluorescence recovery observed in these experiments. To account for these observations, the respective GTPases must cycle within protein complexes at least two orders of magnitude faster than they spontaneously cycle in vitro (see an estimate for cdc42 in Results).

Combining these experimental data with the requirement for the activated form RT suggests that within protein complexes these GTPases cycle both efficiently and rapidly, maintaining a significant excess of the active form while keeping a high turnover rate. This phenomenon is still poorly understood and cannot be explained simply by the relative excess of GEFs over GAPs or by the spatiotemporal segregation of their activity. While separate effects of GEFs and GAPs are thoroughly studied and the kinetic parameters for some well-characterized systems are determined [20–22], the understanding of the interplay of various regulators in the control of GTPase cycling is still lacking. In particular, little is known about the concentrations of GTPases and regulatory molecules that are required to achieve experimentally observed cycling rates and efficiency. The situation is further complicated by the potential involvement of other proteins, such as guanine nucleotide dissociation inhibitors (GDIs) [23] and the GTPase effectors themselves.

In the more developed field of G proteins, a large body of experimental [24–26] and theoretical [27–30] work was done to understand how the amplitude and the duration of G protein signaling are controlled by G protein–coupled receptors (GEFs) and GAPs. This analysis revealed intricate regulatory relationships within the GEF–G protein–GAP module and suggested that variation in the concentrations of its components may define the specific signaling phenotypes [30]. Although G proteins and Ras GTPases are similar in the principle of their operation as molecular switches, they differ in both molecular structure and cellular function. Therefore, it is logical to expect that the control of their cycling would also demonstrate divergent features. Given the critical role that small GTPases play in the regulation of a plethora of cellular functions [31–34], analysis of their control deserves special attention.

Here we use computational modeling to identify regulatory mechanisms that could enable GTPases to cycle with the frequency and efficiency observed in the FRAP-based experiments. We assume a kinetic mechanism in which GTPases cycle while localized on the membrane. For the type of GTPases considered in this work, both RT and RD have high affinity to the membrane; however, the RD form is subject to rapid GDI-mediated membrane–cytoplasm exchange [35]. The counteracting transport processes that depose inactive GTPases on the membrane and return them back to the cytoplasm are thought to be much faster than the rate-limiting GTPase cycling and are not considered here explicitly. Leaving the cytoplasmic GTPase pool and the membrane–cytoplasm transport beyond the scope of the present study, we instead vary the total concentration of the GTPase on the membrane as a model parameter. We also assume that the membrane-anchored GTPases are equally accessible to GEFs and GAPs that are concurrently present on or near the membrane. This mechanism is distinct from the earlier described “Arf-type” mechanism in which only the RT is membrane-bound while RD is strictly cytoplasmic and GEFs are spatiotemporally segregated from GAPs.

Although presently it is not yet feasible to map kinetic mechanisms onto the GTPase families, the mechanism adopted here appears to best describe the cycling dynamics of Rho and Rab GTPases [5,36]. In addition, the majority of the existing experimental data on the rapid cycling of GTPases within protein complexes comes from the same two families. Of these, members of the Rho family, such as cdc42, Rho, and Rac), have been characterized in greater biochemical detail. Therefore, we chose the Rho family of GTPases, in particular its yeast member cdc42p, as a prototype for our model and used the corresponding kinetic parameters to define the model quantitatively (see Methods).

Through a detailed kinetic analysis of the model, we demonstrate that a fast turnover and a high activity of the GTPase can be achieved together only within a specific concentration range of GEF and GAP. The estimated values of these concentrations indicate that the efficient cycling control requires an environment of dense protein complexes. We further extend the analysis by investigating the contribution of a hypothetic scaffold-like GTPase effector that can form a complex with the GEF and amplify the GEF's nucleotide exchange activity while bound to the RT. We demonstrate that such an effector can dramatically enhance GTPase performance and explain the existing experimental data.

## Results

### The GTPase Control Module

**Figure 1:**
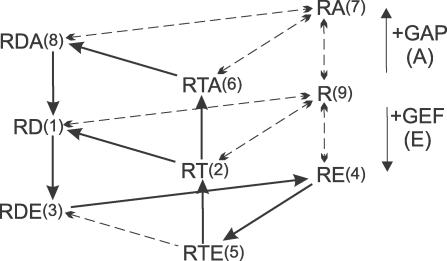
Reaction Scheme of the Small GTPase Cycle The three forms of the GTPase (RD, RT, and nucleotide-free R) (middle layer) form complexes with a GEF (E) (bottom layer) and a GAP (A) (top layer). Solid arrows indicate the main reaction fluxes. All reactions except GTP hydrolyses (2 → 1, 6 → 8, 5 → 3) are reversible. Numbering of the species is consistent with the reaction rate constants listed in Table 3. Free GTP (T), GDP (D), GEF, and GAP are not shown for simplicity.

We developed a mathematical model to describe how the GTPase cycle is regulated by a generic GEF (E) and GAP (A). We reconstructed the core reaction mechanism as shown in Figure 1 on the basis of the analysis of published experimental data. The fundamental GTPase cycle consists of three states: RD, RT, and a short-living nucleotide-free form R. Each of the three states can bind E or A, which catalyze the respective transitions of the GTPase cycle. To simulate the in vivo conditions, concentrations of GTP and GDP were fixed at the levels that are thought to be representative of the intracellular environment (see Methods for details). The resulting reaction scheme of the complete control module consists of 11 species (nine intermediates and free E and A), and their reactions are shown by arrows in Figure 1. The total concentrations of the GTPase, GEF, and GAP, denoted as *R*_0_, *E*_0_, and *A*_0_, are the model parameters that were varied over a broad range to discover all biologically relevant regimes of cycling control.

### GTPases Spontaneously Cycle In Vitro

In the absence of any regulatory molecules, the reaction scheme is given by the middle level in Figure 1 and represents a simple cycle with three states. If the GTP/GDP ratio is taken far from the equilibrium value, e.g., equal to that of a cell, the nucleotides provide a thermodynamic force that drives the GTPase cycle. In the stationary state, all three state fluxes are equal to 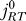, which takes the value 9.67 · 10^−3^ min^−1^ with our model parameters. Further calculations show that the GTPase activity and recovery time in this stationary state are 0.483 and 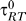 = 35 min, respectively (see Methods for the definitions). Completion of the entire cycle takes 1.5 h with the cycling period *T*_C_ = 100 min. This spontaneous dynamic of a GTPase demonstrates an example of a very inefficient cycling characterized by nearly equal amounts of active and inactive form and a turnover rate incompatible with intracellular processes. Although devoid of biological function, the spontaneous cycling of the GTPase provides a useful baseline for comparison with regulated cycling in vivo.

### GEFs Alone Have Little Effect on GTPase Turnover

**Figure 2:**
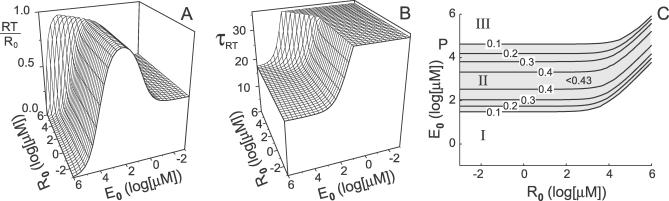
GTPase Cycling in the GEF-Only System The values of the GTPase activity (A), recovery time (B), and performance index (C) are shown as functions of the total concentrations of the GTPase (*R*_0_) and GEF (*E*_0_). The model predicts that in the physiologically relevant range of the GTPase concentrations (*R*_0_ ≤ 10 mM) all three characteristics do not depend on *R*_0_. Roman numerals on the contour plot of the performance index *P* denote three regions with distinct GTPase cycling regimes: (I) effectively control-free, (II) processive (*P* > 0.1, highlighted in light grey), and (III) futile cycling within the complex with the GEF. Numbers on the contour lines indicate the levels of *P*.

We first asked how a single GEF, unaided by other regulatory molecules, influences GTPase cycling. The reaction scheme in this partial control scenario is represented by the middle and bottom levels of the complete diagram in Figure 1. To address the above question, we analyzed model behavior in a broad range of GTPase and GEF concentrations (1 nM–1 M) (Figure 2). At a constant *R*_0_, GTPase activity shows a characteristic biphasic response to the increase in the GEF concentration (Figure 2A). First the activity rises, starting from the level of the control-free regime 0.483, reaches its maximum 0.958 at *E*_0_ = 250 μM, and subsequently decreases to zero. A detailed analysis of the reaction fluxes shows that the proportion of the GTPase that cycles without dissociation from the GEF continuously increases as *E*_0_ grows. This GTPase fraction becomes trapped in a futile cycle 3 → 4 → 5 → 3 at the bottom of the reaction scheme and no longer participates in the processive cycle 1 → 3 → 4 → 5 → 2 → 1. This explains the decrease in the apparent GTPase activity with the increase in *E*_0_. Surprisingly, the model predicts that in the physiologically relevant range of GTPase concentrations (up to ∼10 mM), the profile of the GTPase activity does not depend on *R*_0_. An important consequence of this prediction is that the same, fairly high concentration of the GEF is necessary to maximize the activity of the GTPase regardless of its total concentration.

The profile of the performance index *P* shown in Figure 2C highlights the existence of three distinct regimes of the GTPase cycling: (I) effectively control-free regime with the main cycle 1 → 9 → 2 → 1, (II) processive regime 1 → 3 → 4 → 5 → 2 → 1, and (III) GEF-chaperoned regime 3 → 4 → 5 → 3. Only within the intermediate processive regime is the cycling control efficient and does it result in a significant increase in GTPase activity. At the same time, the turnover rate of GTPase in the GEF-only system is at most twice that of the control-free GTPase. As follows from Figure 2B, the GTPase recovery time is bounded between the control-free value of 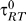 = 35 min and the minimum of 17 min that is reached at saturating *E*_0_. This result demonstrates that the experimentally observed rates of GTPase turnover cannot be explained solely by the activity of GEFs.

### GAP Is Necessary for the High GTPase Turnover Rate

**Figure 3:**
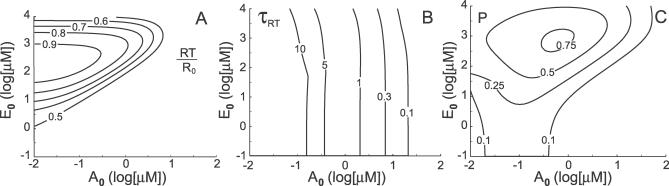
Concentrations of GEF and GAP Define the Efficiency of the GTPase Cycling The profiles of the GTPase activity (A), recovery time (B), and performance index (C) are plotted as contour lines. While activity remains largely a function of the GEF concentration, the recovery time is determined mostly by the GAP concentration. Performance maximum with the activity 85% and recovery time ∼3 min is attained at *E*_0_ = 0.78 mM and *A*_0_ = 0.66 μM. Further increase in the turnover rate can be achieved at the expense of activity. Numbers on the contour lines indicate the levels of the respective characteristics. Total GTPase concentration is *R*_0_ = 1 mM.

Introduction of a GAP into the reaction scheme brings about profound changes in the operation of the GTPase control module. Simulation of the complete GEF–GAP reaction scheme reveals a regime with high GTPase activity at large GEF and small GAP concentrations (see Figure 3A). Comparison with Figure 2A shows that the addition of a GAP results in the expected erosion of the activity peak and its shift toward higher *E*_0_ as *A*_0_ increases.

Strikingly, the model predicts that the GTPase turnover rate quantified by the recovery time τ*_RT_* is almost exclusively a function of the GAP concentration (Figure 3B). τ*_RT_* exhibits some dependence on *E*_0_ only at very large GEF concentrations, which are likely beyond the physiologically relevant range. This result suggests that GAP is the main factor that determines the observed in vivo fast turnover rates of the GTPase-controlled complexes.

The profile of the performance index (Figure 3C) has a single broad maximum centered at *E*_0_ = 0.776 mM and *A*_0_ = 0.66 μM. At these optimal concentrations of GEF and GAP, the recovery time, τ*_RT_* = 3 min, is more than ten times shorter than in the control-free system and is accompanied by a high activity of 0.85. Further reduction in the recovery time can be achieved by increasing the GAP concentration, however, at the expense of GTPase activity.

Importantly, we find that profiles of activity, recovery time, and performance index are virtually insensitive to the concentration of the GTPase itself. At *R*_0_ = 1 μM, which is three orders of magnitude lower than the total concentration of the GTPase in Figure 3, the maximum of *P* shifts to 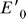 = 0.631 mM and 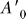 = 0.19 μM, while the values of all performance characteristics remain essentially the same.

### In the Efficient Regime, the GEF–GAP Control Module Can Be Reduced to a Single Cycle

In search for the mechanism that provides an increased efficiency and cycling rate in the GEF–GAP system, we analyzed the reaction fluxes at varied concentrations of GEF and GAP. We noticed that at large *E*_0_ and *A*_0_, only a subset of fluxes (shown by thick solid arrows in Figure 1) is significant, while the remaining fluxes have negligible values. To test the hypothesis that only the main fluxes are important for efficient functioning of the GTPase control module, we introduced a reduced model in which only the subset of significant fluxes was retained. Analysis of the complete model showed that it always possesses only one stable steady state and thus we do not risk losing a steady state by reducing the complete model. Rigorous comparison of the complete and reduced models demonstrated that in the (*A*_0_, *E*_0_) domain with efficient cycling control, where both the GTPase activity and the turnover rate are high, the flux vectors of the reduced and complete reaction schemes differ by less than 5% (see Methods). The difference between the two models continuously diminishes as the total concentrations of GAP and GEF increase. Neglecting the fluxes, which are nonessential at large *E_0_* and *A_0_*, results in a simple, biologically intuitive reaction scheme. Thus, the reduced model shows that in the efficient regime the operation of the GEF–GAP control module is based on two linear pathways: the GEF arm 1 → 3 → 4 → 5 → 2 and the GAP arm 2 → 6 → 8 → 1. These pathways catalyzed by the GEF and GAP effectively short-circuit the nonessential parasitic reactions that slow down the GTPase cycling. The cycle formed by the union of the two pathways is, in fact, the fastest GTPase cycling loop with the period *T_C_* = (*k*_13_ *E*)^−1^ + (*k*_26_ *A*)^−1^ + 0.2 min, where the constant term is the sum of the inverse first-order rate constants in the cycle. Note that the actual value of *T_C_* is a function of concentrations of the free GAP and GEF, which grow together with the respective total concentrations.

The reduction of the model to a single loop allowed us to derive some analytical estimates for the activity and recovery time (see Methods for derivation). These estimates are useful for the interpretation of the above results. Thus, a straightforward calculation shows that the GTPase recovery time is a sole function of the free GAP concentration

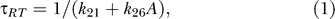
in complete agreement with the simulation results. Comparison of the numerically simulated and analytically computed recovery times (see Methods) shows that this estimate for τ*_RT_* is valid in a very broad range of (*A*_0_, *E*_0_), the total concentrations of GAP and GEF, respectively. We also derived an estimate for the GTPase activity

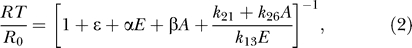
which closely approximates the numerically simulated results and explains the biphasic dependence of the activity on the concentration of GEF (see Methods for definitions of constants). Comparison of the two derived estimates highlights the nature of the conflict between the activity and the turnover rate. While a decrease in τ*_RT_* requires a larger *A,* an increase in the activity demands a lower free GAP concentration.

### Scaffold Effector Can Significantly Boost the Performance of GTPase Cycling

Comparison of the above results with the experimental data suggests that additional levels of control may be involved to enable the observed rapid cycling rates without a significant loss in GTPase activity. Potential candidates for this role are GTPase effectors that bind a GEF and increase its activity. Indeed, in a number of molecular modules reported in recent literature, a scaffold-like effector has been found in the complex with the active GTPase and its GEF [4]. Importantly, for some of these effectors, such as intersectin-l [37] and Rabaptin-5 [38], it has been quantitatively shown that, as long as the effector stays in complex with the RT, the activity of the bound GEF increases several fold. For further analyses we assumed that scaffold-effector S forms a stable complex E^*^ with the GEF E at all times [39,40]. Binding of the E^*^ to the RT results in the formation of an activated tripartite complex M (RT-S-E) whose activity is substantially higher than that of the original GEF E. A detailed kinetic analysis of the complete reaction scheme that includes all possible reactions of the complex E^*^ would require the introduction of nine intermediates (·)E^*^(·), where (·) stands for any of the RD, RT, or R, and the knowledge of an additional 54 reaction rate constants. Since at the present moment none of the known scaffold-effector systems has been characterized at this level of kinetic detail, we resorted to a coarse-grained modeling approach that requires only three additional rate constants. Based on the experimental data for several GTPase effectors [41,42], we estimated the rate constants for the formation *k*_2M_ = 600 μM^−1^min^−1^ and dissociation *k*_M2_ = 18 min^−1^ of M (*M* ↔ *RT* + *E* ^*^) and represented the activity of M as a single catalytic step *RD* + *M* → *RT* + *M* with effective second-order rate *k*_12_ = 0.6 μM^−1^min^−1^.

**Figure 4:**
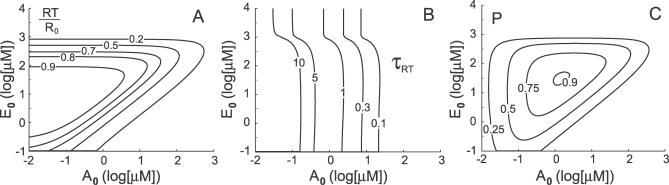
The GTPase Activity and the Overall Cycling Performance Are Improved by the Introduction of a GEF-Activating GTPase Effector S The profiles of the GTPase activity (A), recovery time (B), and performance index (C) are plotted as contour lines. Note the increase in the area of the optimal control and its shift toward lower GEF concentrations in comparison with Figure 3C.

Simulation of our model demonstrates that scaffold-effector S significantly increases the performance of the GTPase control module. Comparison of Figures 3 and 4 shows that, while the recovery time does not change noticeably, the GTPase activity is considerably enhanced. This enables a high GTPase activity at the large GAP concentrations where the recovery time is short. For example, at 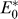 = 100 μM and *A*_0_ = 40 μM, the model predicts the GTPase activity 0.72 and the recovery time 3.2 s.

Importantly, our model suggests that the involvement of a GEF-activating scaffold-effector substantially reduces the optimal concentration of a GEF. Thus, the maximum of the performance index is located at 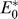 = 27.9 μM, which is more than 20 times smaller than the corresponding value in the system without M. In a stark contrast with the GEF–GAP module, performance of the M–GAP system scales with the total concentration of GTPase. As follows from Table 1, the values of the activity and recovery time computed at the maximum of the performance index improve with an increase in *R*_0_.

**Table 1:**
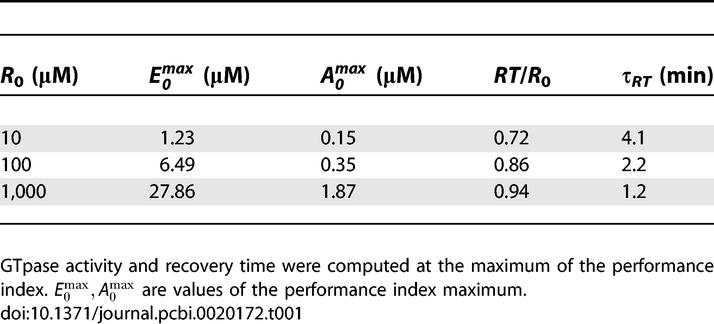
Performance of the M–GEF Control Module with the Scaffold-Effector Depends on the Total GTPase Concentration

## Discussion

Here we quantitatively investigated how the cycling dynamics of a small GTPase are controlled by GEFs and GAPs. Although our model is predominantly based on the Rho family of GTPases, it is mechanism- rather than molecule-specific. Under the assumptions of our model, GTPases can freely cycle and form complexes with effectors and regulatory GEFs and GAPs while remaining anchored to the membrane. According to the existing FRAP data, this “Rho-type” mechanism is exhibited by at least some members of the Rho and Rab families, such as cdc42 [13], Rac2 [19], and Rab5 [18]. Future research will undoubtedly identify more small GTPases that follow this mechanism as well as likely reveal more functional patterns of GTPase cycling, distinct from both “Arf”- and “Rho”-type mechanisms.

To elucidate the individual roles of regulatory proteins as well as their synergistic action, we compared several possible designs of the control module with progressively increasing complexity. Characterizing the stationary activity of the GTPase and its turnover rate as either high or low, one can distinguish up to four qualitatively distinct regimes of cycling. Of these we concentrated on the regime with high activity and high turnover rate since this is the only regime that is relevant for the rapid recovery of fluorescence observed in the FRAP-based studies described here. The fundamental question that we sought to address is how GTPase cycling is controlled so that high activity is achieved in parallel with a rapid turnover.

A convenient baseline for this analysis is provided by the spontaneous cycling of a GTPase in a chemostat with the intracellular concentrations of GTP and GDP. Under these control-free conditions, yeast cdc42p would cycle with the basal activity of ∼0.5 and the turnover rate nearly three orders of magnitude lower than the rate observed in the emerging bud [13]. In vivo, this slow “parasitic” cycling is thought to be prevented by GDIs that sequester the inactive form of the GTPase and block it from shedding the GDP [43]. Several lines of evidence suggest that GDIs play an important role in the transport of GTPases between cellular compartments [35]. However, once a GTPase molecule is localized on the membrane, its cycling is not appreciably affected by GDIs [43], and GDI knockouts often show no changes in GTPase functions [35,44]. Since transport processes are left outside the scope of the present analysis, we did not consider the influence of GDIs on GTPase cycling.

Analysis of the three plausible designs of the cycling control module represented by a single GEF, a GEF–GAP module, and its modification with a GEF-activating GTPase effector have revealed a number of common trends. We demonstrated that GEF and GAP play distinct and separable roles in cycling control. While the activity of GTPase in a stationary state is mainly defined by the activity of the GEF, the turnover rate, which is inversely proportional to the GTPase recovery time, is almost entirely a function of the free GAP concentration. Our model shows that in the absence of a GAP, GEF alone can only marginally increase the turnover rate. Combined action of GAP and GEF amplifies the turnover of the GTPase and reduces the recovery time by at least one order of magnitude. The involvement of a scaffold-effector that increases the activity of a GEF can significantly improve the GTPase cycling performance by boosting both the activity and turnover rate. This additional level of control reduces the recovery time by another order of magnitude, bringing it into the range reported in FRAP experiments.

In the field of G protein signaling it has been observed that GAPs accelerate the termination of signaling without reduction of the signal amplitude [24,25]. We found that the maximization of the activity and turnover rate of a small GTPase impose conflicting requirements on the GAP concentration. While the activity is always reduced by the GAP, the turnover rate grows proportionally to the GAP concentration. Therefore, to achieve a high activity and turnover rate at once, the concentrations of GEF and GAP should be tightly controlled to remain within the optimal range. Based on the contradictory nature of the requirement for the GAP, we predict that future experiments will reveal two distinct subclasses of the GTPase-controlled systems: optimized for high turnover rate and for high activity. Cycling regimes of a small GTPase are found to be less dependent on its total concentration than the activity of G proteins on the G protein concentration [30]. For the GEF–GAP module, our model predicts that 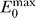 and 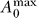 , the optimal total concentrations of GEF and GAP, are almost insensitive to the variation of the GTPase concentration, *R*_0_. As *R*_0_ increases by three orders of magnitude, 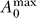 modestly rises ∼3.5-fold, while 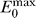 remains virtually the same. On the contrary, the performance of the modified M–GAP module steadily increases with the GTPase concentration (Table 1). This difference can be potentially utilized to experimentally distinguish the two designs of the control module.

Similar to the control of G protein signaling [30], concentrations of GAP and GEF were identified as the major factors that determine the character of the cycling regime. Our results indicate that for the GEF–GAP module to provide acceptable GTPase activity and turnover rate, the total concentration of the GEF should be on the order of 1 mM. Such high concentrations are possible only within dense protein clusters assembled on the membrane. For comparison, a compact hexagonal packaging of spherical protein complexes with diameter 10 nm would result in an effective 1.93 mM concentration of its components in a 10-nm-thick layer of the cytoplasm above the membrane surface. Reduction of this concentration requirement by an effector-scaffold, which was demonstrated here for the M–GAP module, allows for sparser clusters. It is also tempting to speculate that for certain low-abundant GEFs, the co-option of such effectors may provide a vital mechanism that enables effective control over the respective GTPases despite a low cellular copy number. These hypotheses may offer some insight into why GEF-effector complexes are increasingly found as a recurring motif of the complex-formation control modules [4]. Thus, apart from the quantitatively characterized cases of intersectin-l and Rabaptin-5, the increase in the GEF activity has been observed in the yeast complexes Sec4p[GTPase]-exocyst[effector]-Sec2p[GEF] [45] and Ypt7p[GTPase]-Vps/HOPS[effector]-Vps39p[GEF] [46,47]. Although all GTPases in the above examples belong to either Rho or Rab families, the phenomenon of a GEF-activating GTPase effector is likely not restricted to these two families only. Thus, Sos is simultaneously an effector and a GEF for Ras GTPase [48]. In this peculiar case, the roles of the effector and the GEF are performed by two different domains of the same protein. It is conceivable that Sos evolved as a fusion of two originally independent proteins. Regardless of the molecular implementation, the function of all GEF-activating effectors is to provide a positive feedback loop that increases the cooperativity of GTPase activation.

Our results indicate that maintaining optimal concentrations of regulatory proteins in the focus of complex formation is the key factor for achieving efficient GTPase cycling. Since these concentrations are typically several orders of magnitude higher than the respective cytoplasmic concentrations, powerful mechanisms must be in operation to recruit regulatory molecules to the membrane. Several candidate mechanisms based on protein–lipid [49,50] and protein–protein interactions [51,52] have been identified and require further investigation. These mechanisms often form the basis for positive and negative regulatory feedback loops [53,54] and are likely to increase the cooperativity of protein complex formation. The integration of these upstream mechanisms with the GTPase control module will result in more realistic models of GTPase-controlled complex formation and bring a better understanding of a great variety of cellular processes that depend on cycling dynamics of small GTPases.

## Methods

### Model formulation and rate constants.

We formulated the chemical kinetics corresponding to the scheme shown in Figure 1 as a system of 11 mass-action rate law equations with 27 rate constants. For example, the equation describing the time evolution of RD is

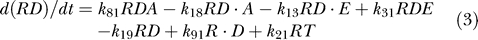



Detailed formulation of each model equation is given in Table 2, which also contains an equation for M, the complex of RT and E^*^. Concentrations of nucleotides were considered constant and equal to the average cell concentrations typically reported in the literature [55]: 500 μM for GTP (T) and 50 μM for GDP (D). Depending on the type and state of the cell, these concentrations may vary and cause a slight alteration of all reaction fluxes since the thermodynamic force that drives GTPase cycling depends on the logarithm of the GTP/GDP ratio [56]. However, if such a variation is within biologically meaningful limits and does not lead to the breakdown of cellular homeostasis, it does not affect any of the qualitative conclusions of this study.

**Table 2:**
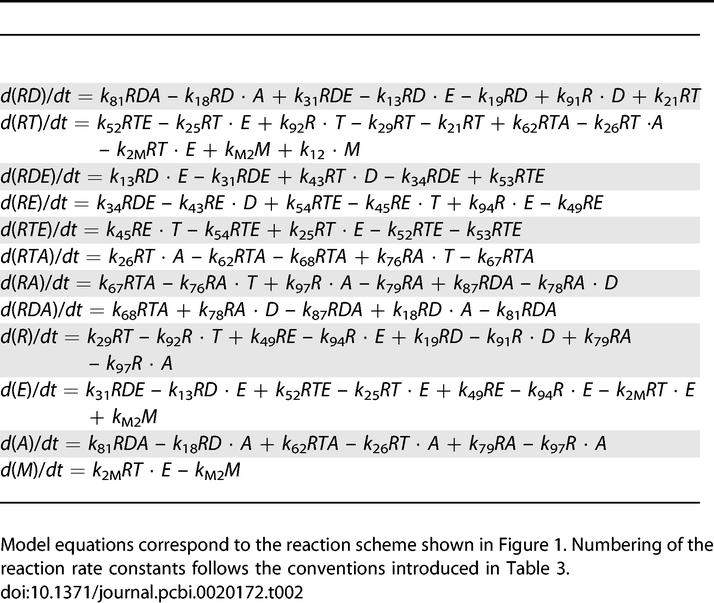
Model Equations

**Table 3:**
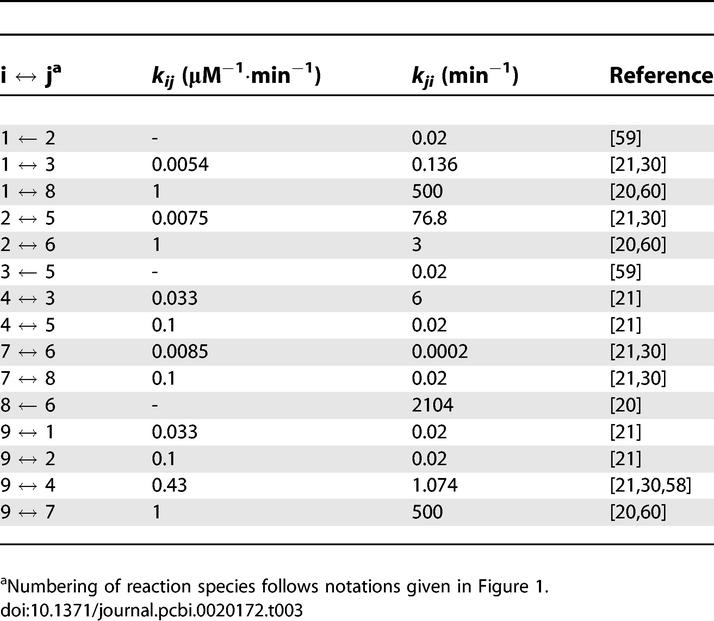
Reaction Rate Constants Used in the Model Simulations

**Figure 5:**
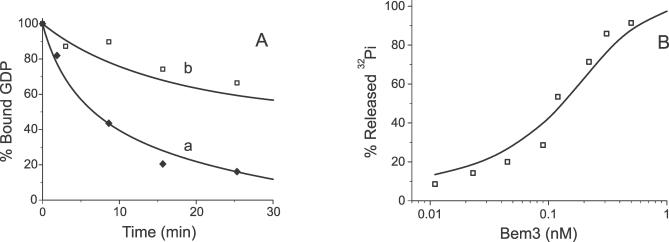
Simulation of the Yeast cdc42p Cycling In Vitro (A) Kinetics of GDP dissociation from [^3^H]GDP-bound cdc42p after mixing with GTP. Comparison of the model simulations and experimental results [59] is shown as groups **a** and **b** for the reactions with and without GEF cdc24p, respectively. Concentrations of cdc42p, cdc24p, and GTP were 90 μM, 28 μM, and 100μM. (B) Simulation of GAP-stimulated GTP hydrolysis by cdc42p. Experimental data are for the yeast GAP Bem3 [60]. GTP hydrolysis was essayed by measuring phosphate (Pi) release over 5 min. Initial concentration of [γ-^32^P]GTP-bound cdc42p was 0.1 μM. Simulation results are shown by solid lines.

Where available, rate constants were derived from the in vitro data for the yeast cdc42p. Remaining constants were estimated from the published experimental data on other small GTPases of the Rho family. Rate constants were further constrained by fitting the model to the available data for the yeast cdc42p (Figure 5). The values of the rate constants together with their sources are summarized in Table 3.

### Reaction fluxes.

**Figure 6:**
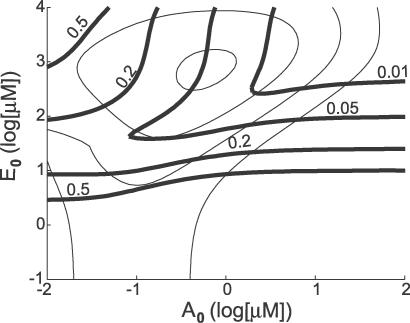
Comparison of the Complete and Reduced Models for the GEF–GAP Cycling Control Module The Euclidean distance between the flux vectors of complete and reduced models was computed as described in Methods and normalized by the magnitude of the flux vector for the complete model. The profile of the normalized distance is shown by thick solid contour lines. The profile of the performance index *P* (same as Figure 3C) is shown by a thin line for comparison. Note that in the parameter region where the GTPase cycles with a high activity and turnover rate, the difference between the complete and reduced models is 5% or less.

Starting from arbitrary initial conditions, the reaction scheme in Figure 1 relaxes to a stationary state characterized by a time-independent set of stationary concentrations and generally nonzero reaction fluxes. A transition reaction flux *J_lm_* that connects species *l* and *m* can be readily calculated from the species concentrations and the rate constants, e.g., flux *J*_13_ connecting RD and RDE (see Figure 1) is

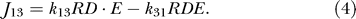
The sign of *J_lm_* defines the flux direction. For each node *i* of the reaction scheme, we can compute the influx 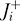 , the sum of all transition fluxes that enter the node, and the efflux 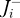 , the sum of all outbound fluxes. In the stationary state, both fluxes become equal 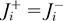 = *J_i_*. The state flux *J_i_* is, thus, the stationary flux through the node *i*. To compensate for the dependence of all reaction fluxes on the system size, we normalized all flux values by the total concentration of the GTPase, i.e., *j*_i_ = *J*_i_/*R*_0_. The reaction scheme with *n* transition fluxes *j_lm_* can be represented by the flux vector 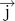 in the *n*-dimensional space. The Euclidean distance between two flux vectors 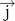 ^1^ and 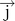 ^2^ as defined by

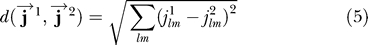
can be used, for example, as a measure of difference between two states of a reaction scheme. If 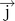 ^2^ is taken as a reference flux vector, division of *d *( 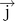 ^1^, 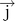 ^2^) by the Euclidean norm of 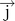 ^2^ will give a relative displacement of 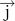 ^1^ from 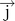 ^2^ in a nondimensional form. We used the normalized Euclidean distance to measure the deviation of the flux vector of the reduced model from that of the complete model (see Results). Figure 6 demonstrates that in the parameter region characterized by efficient GTPase cycling, the difference between the complete and reduced models is less than 5%.

### Characteristic times.

For each reaction *l* → *m* it is possible to define a mean transition time *t*_lm_ = 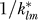 that a molecule spends on average in the transition from node *l* to *m,* where 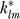 is the reaction rate *k*_lm_ for the first-order reactions and is equal to *k*_lm_ *X* (*X* = *A, E, D, T*) for the second-order reactions [56], e.g., for the chosen earlier example *t*_13_ = 1/(*k*_13_ *E*) and *t*_31_ = 1/*k*_31_. Adding up the transition times along any closed loop in the reaction scheme, we obtain a *cycling period T*_C_ that is required on average to complete the full cycle over the chosen loop. In a stationary state, molecules of each species are continuously synthesized and consumed while their concentrations remain constant. To characterize the time that is necessary for the system in a stationary state to turn over half of the molecules in the node *i,* we define a *recovery time*:

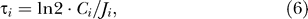
where *C*_i_ and *J*_i_ are the stationary concentration and the state flux, respectively. This introduced recovery time is directly comparable with the half-life time measured in the FRAP experiments.

### Performance characteristics.

To quantitatively describe the efficiency of the GTPase cycling control in a stationary state, we computed the fractional GTPase activity *RT*/*R*_0_ (in the text it is “activity” for brevity) and the recovery time τ*_RT_* of the GTPase. Since efficient control is expected to optimize both the activity and the turnover rate, we introduced a single nondimensional performance index:

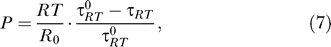
where 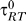 is a reference recovery time taken to be equal to the recovery time calculated for the spontaneous cycling of the GTPase in vitro (see Results).

### Derivation of the analytical estimates for the GTPase activity and recovery time.

**Figure 7:**
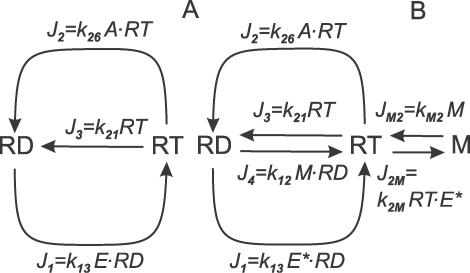
Flux Diagrams for the Reduced Models of GEF–GAP (A) and M–GAP (B) Modules Activation of RD by the GEF or by the GEF-effector complex (E^*^) is denoted as *J*_1_. Inactivation of RT by the GAP and its spontaneous hydrolysis are represented by fluxes *J*_2_ and *J*_3_, correspondingly. Activation of RD by M is represented by *J*_4_ in (B).

Reduction of the GEF–GAP control module to the subset of significant fluxes results in a dramatic simplification of the model and allows us to estimate the GTPase cycling characteristics analytically. As shown in Figure 7, the entire system of reaction fluxes is reduced to three main fluxes:

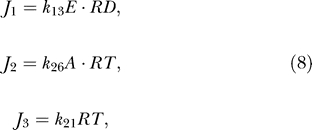
where *J*_1_ is the flux through the GEF arm (1 → 3 → 4 → 5 → 2), *J*_2_ is the flux through the GAP arm (2 → 6 → 8 → 1), and *J*_3_ describes spontaneous transition of RT into RD. From the balance of fluxes in a stationary state, it follows that the stationary flux through RT is given by:

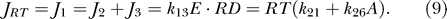



Rearranging the terms, we find the ratio of the active and inactive forms of the GTPase in a stationary state:

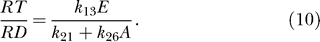



Now, from the definition given above, it immediately follows that the recovery time of the GTPase can be expressed as:

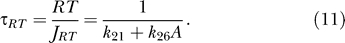



Thus, in the parameter domain (*E*_0_, *A*_0_), where the reduction of the flux system is valid, the recovery time is a sole function of the free concentration of the GAP.

Using the reduced reaction scheme shown in Figure 7A, we also can estimate the activity of the GTPase. Inspection of the simulation results shows that in the parameter domain of interest, the prevalent forms of the GTPase are RT, RD, RDE, and RTE, while the others are practically negligible. In this case, the activity can be defined as:

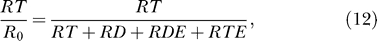
where RDE and RTE can be expressed through the stationary concentrations of RT and RD, using the relationships between the reaction fluxes and species concentrations. For example, since

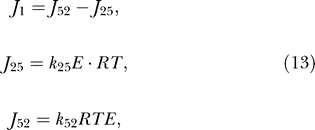
we can express the stationary concentration of the RTE intermediate complex as:

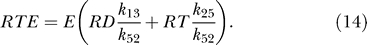



In a similar way, *RDE* = *k*_13_/*k*_34_ *RD* · *E*. Assembling all terms and using the expression for the ratio of RT and RD, we find an estimate for the GTPase activity:

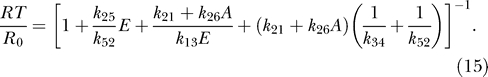



Introducing the notations:

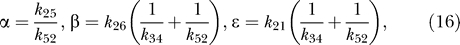
we finally obtain the activity in the form presented in Results:

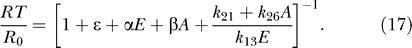



**Figure 8:**
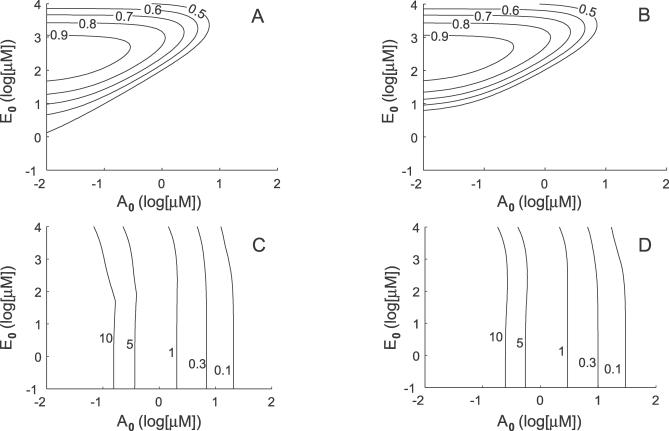
Comparison of the Numerically Computed and Analytically Estimated Cycling Characteristics The activity (upper row) and the recovery time (bottom row) profiles of GTPase are plotted versus the total concentrations of GEF and GAP. Results shown in (A) and (C) were numerically simulated using the complete model, while those shown in (B) and (D) were calculated based on the analytical estimates derived from the reduced model. Note the good match between the simulated and analytically estimated results in a broad domain of parameter values.

Figure 8 shows that the derived analytical expressions for τ*_RT_* and *RT/R*_0_ correspond well with the numerically simulated values in a broad domain of parameter values.

Similar estimates can be derived for the M–GAP system where scaffold-effector S forms a stable complex E* with the GEF E. As follows from Figure 7B, in a stationary state, the concentration of M is in a simple relationship with that of the RT:

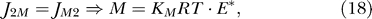
where *K_M_* = *k*_2*M*_/*k_M_*_2_ is the binding constant of RT to M. The introduction of M results in an additional flux *J*_4_ = *k*_12_ *RD* · *M* that acts in parallel with *J*_1_ = *k*_13_ *RD* · *E* ^*^. Since in a stationary state essentially all E* is converted to M, *E* ^*^ << *M* and *J*_1_ << *J*_4_. From the balance of fluxes,

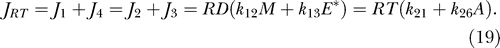



Neglecting *J*_1_ in comparison with *J*_4_ and using the expression for *M*, we find that

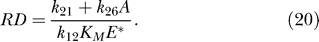



This estimate can be used to find the recovery time of the GTPase

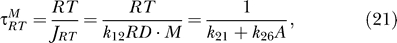
which is identical to the recovery time in the GEF–GAP module. This result emphasizes that the recovery time is defined by the activity of the GAP alone and, thus, is not affected by the introduction of the scaffold-effector S.

Along the same lines, the activity of the GTPase in the M–GAP module can be expressed as:

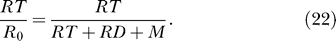



From the balance of GEF complexes 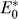 = *E* ^*^ + *M* + *ɛ,* where *ɛ* stands for the negligible concentrations of all the other species (e.g., RDE*, RTE*, RE*), we express RT as a function of 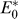 and *E* ^*^ as follows:

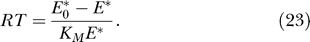



After replacing RT, RD, and M with the corresponding expressions, we obtain the sought-after estimate for the activity of the GTPase in the M–GAP module:

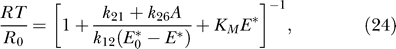
whose form is analogous to that for the GEF–GAP system.

### Computational implementation.

All numeric simulations and analysis were performed with Matlab (MathWorks, http://www.mathworks.com), which was equipped with the Systems Biology Toolbox [57].

## Supporting Information

### Accession Numbers

National Center for Biotechnology Information (http://www.ncbi.nlm.nih.gov) Entrez Protein accession numbers are: cdc42 (AAB40051), cdc42p (NP_013330), CNK1 (AAH12797), COP I (NP_009194), COP II (NP_057535), GAP Bem3 (NP_015210), GEF cdc24p (NP_009359), cdc42p (NP_01333), intersectin-l (NP_003015), Rab5 (AAH09823), Rabaptin-5 (CAA62580), Rac (CAB53579), Rac2 (AAB20524), Ras GTPase (NP_476699), Rho (AAH01360), Sec4p[GTPase]-exocyst[effector]-Sec2p[GEF] (NP_014127), Sos (NP_476597), WASP (AAA62663), and Ypt7p[GTPase]-Vps/HOPS[effector]-Vps39p[GEF] (CAA48244, BAA11758).

## Abbreviations

GAP: GTPase activating protein
GDI: guanine nucleotide dissociation inhibitor
GEF: guanine nucleotide exchange factor
RD: GDP-bound GTPase
RT: GTP-bound GTPase
