# Correction: Computational Model Explains High Activity and Rapid Cycling of Rho GTPases within Protein Complexes

**DOI:** 10.1371/journal.pcbi.0030136

**Published:** 2007-07-27

**Authors:** Andrew B Goryachev, Alexandra V Pokhilko

In *PLoS Computational Biology*, volume 2, issue 12: doi:10.1371/journal.pcbi.0020172

In Table 2, the first line was missing the last term: −*k*_12_ *RD·M*. The same term was incorrectly presented in the second equation: +*k*_12_ *·M* (the "RD" was missing). In the third line, the term *k*_43_ *RE·D* was incorrectly presented as *k*_43_ *RT·D*. The correct Table 2 is below.

**Table 2:**
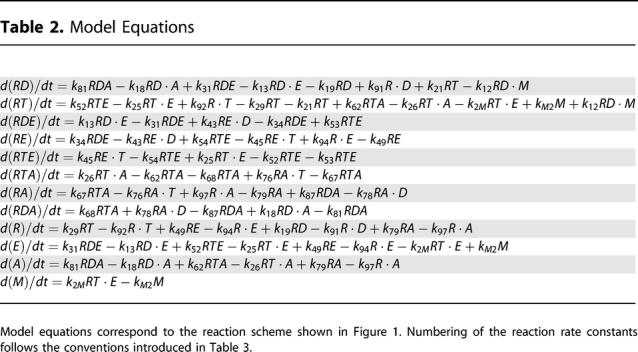
Model Equations

